# Response Bias by Neuroblastoma Screening Participation Status and Social Desirability Bias in an Anonymous Postal Survey, Ishikawa, Japan

**DOI:** 10.2188/jea.11.70

**Published:** 2007-11-30

**Authors:** Kiyoko Makimoto, Yoshie Iida, Masao Hayashi, Fumie Takasaki

**Affiliations:** 1 Osaka University, Department of Nursing, Osaka, 565-0871, Japan.; 2 Health Promotion Division, Department of Health and Welfare, Ishikawa Prefecture, Japan.; 3 Kanazawa University, Japan.

**Keywords:** response bias, social desirability bias, screening, postal survey

## Abstract

Objective: To examine response bias by neuroblastoma screening participation status in a population-based postal survey of parents in Ishikawa Prefecture, Japan

Methods: The eligibility criteria for the study were: 1) parents whose infants were born in Ishikawa Prefecture between March 1997 and February 1998, and 2) of those parents who resided in the Prefecture in March 1999. Four-page questionnaires were mailed to one-third of screening participants (n= 2,886) and all the nonparticipants (n= 1,401). Questionnaires were anonymous, with no identifiers on the questionnaire. Colored papers were used for printing questionnaires to differentiate screening participation status. Response rates were calculated using demographic information on the infant registry as the denominator and demographic characteristics data from the returned questionnaire as the numerator.

Results: The response rate was 63% for participants and 33% for nonparticipants. The following factors were associated with lower response rates regardless of screening participation status: older maternal age (≥35 years), higher parity (≥4), nuclear family status, and mother having a full-time occupation. Approximately 20% of screening nonparticipants reported having participated in the screening. Place of residence, maternal age, and parity were associated with the percentage of incorrect reporting.

Conclusion: Screening participation status was a major factor associated with low response rate, although some demographic characteristics were also predictive of low response rates. Incorrect reporting of screening participation among nonparticipants indicates a strong social desirability bias in this official survey in Japan.

## INTRODUCTION

A neuroblastoma screening program was instituted in Japan in 1985. The screening is offered free of charge by the Prefectural government when parents bring their children for health and developmental check-ups at 3 months of age. Municipal offices provide a container, an addressed envelope, and a leaflet explaining how to collect a urine sample. Parents are requested to send the specimen to the laboratory when the child reaches 6 to 8 months of age. Each municipality sends the Public Health Department (PHD) a list of infants and their addresses. The five PHDs maintain a neuroblastoma screening registry, which includes mother’s demographic information, screening participation status, and lab results. The main laboratory sends the results to the PHDs, and the PHDs notify parents if further tests are necessary.

Currently, the effectiveness of the neuroblastoma screening program is being questioned^1^^)^, and a large scale cohort study is being conducted to evaluate the screening program in Japan. In Ishikawa Prefecture, a few neuroblastoma cases among screening nonparticipants came to attention in recent years. Then, the department of health and welfare planned a postal survey to improve the screening process and participation rates before the efficacy of neuroblastoma screening was questioned in Japanese medical society. Parents had not been notified about the controversy over the efficacy of screening. This survey provided an opportunity to study response bias in a population-based survey of parents by infant screening participation status.

Response bias for postal survey has been examined mainly in European countries and in the United States^2^^-^^4^^)^. Studies found that nonrespondents generally differ in terms of demographic characteristics, functional status, and health conditions. It is difficult to detect response bias in an anonymous survey by respondents’ characteristics. The current survey used colored papers to differentiate screening participation status on the returned questionnaire. The purpose of the study is to examine response bias in an anonymous survey by screening participation status.

## MATERIALS AND METHODS

The eligibility criteria for the study were: 1) parents whose infants were born in Ishikawa Prefecture between March 1997 and February 1998, and 2) of those parents who resided in the Prefecture in March 1999. Screening participant is defined as the parent whose infant has had lab results recorded on the registry; nonparticipant is defined as the parent whose infant has not had lab results recorded. The number of eligible neuroblastoma screening participants was 9,685, and the number of eligible nonparticipants was 1,401 in the prefecture at the time of the survey. All the nonparticipants were included in the study, and about one-third of the participants were chosen using systematic sampling to select every third participant on the list within each PHD.

The four-page questionnaire included 22 items. Twelve items solicited demographic information and screening participation status; 6 items for screening participants asked reasons for participation and problems associated with screening procedures; 4 items for nonparticipants asked reasons for nonparticipation. The questionnaire also requested the following demographic information: family structure, birth order of the infant, maternal age at the time of the survey, and maternal occupation when the infant was 6 to 8 months old. The questionnaire with a cover letter was mailed in March 1999, and those returned within three weeks were included in the analysis.

This was an anonymous postal survey, and colored papers were used to print questionnaires in order to distinguish screening participation status (white for participants and light blue for nonparticipants).

The response rate was calculated by PHD, and the response ratio was calculated by demographic characteristics. The term ratio was used because the survey data could not be linked to the screening registry, and numerators may not arise from denominators due to missing data or inaccurate reporting. The data on the screening registry were used as denominators, and the data on the returned questionnaire were used as numerators. One year was subtracted from the maternal age on the questionnaire because of the time lag between screening and the survey. No reminders were sent because of budget constraints.

## RESULTS

The total number of questionnaire filled and returned was 2,285, and the number of returned questionnaire with unknown address was 9. The response rate was 63% for participants and 33% for nonparticipants, and rates varied little among the five PHDs (Table 1).

**Table 1.** Response rates by public health district and screening participation status.

Public Health Districts	Urban/rural classification	Participants		Nonparticipants		Overall rates*
		N	%	N	%	%
Kaga	Urban/rural	575	62	279	32	58
Ishikawa-Central	Suburban	572	60	267	33	56
Noto-Central	Rural	319	64	155	34	60
Noto-North	Rural	160	68	81	32	63
Kanazawa	Urban	1260	65	619	32	60
Total		2886	63	1401	33	59

*Overall rates were adjusted for sampling.

The mean maternal age was 29.8 ± 4.2 years. Forty-four percent of mothers were primiparous, and mothers having three or more children accounted for 19% of the sample. Full-time housewives comprised 60% of the sample, and mothers with full-time employment accounted for 28%. The majority of the families were nuclear families, and extended families comprised over 30% of the sample.

### Response ratios by demographic characteristics and screening participation status

Response ratios varied by parity, maternal age, maternal occupation, and family structure (Table 2).

**Table 2.** Postal survey response ratios by neuroblastoma screening participation status and demographic characteristics, ishikawa prefecture, Japan, 1999.

		Participants		Nonparticipants	
		N	%	N	%
Parity	1	1407	65	489	36
	2	995	64	576	31
	3	368	64	239	34
	≥4	116	33	97	21
	Missing	0	N/A	0	N/A

Maternal age	≤24	208	87	201	35
	25-29	1085	73	530	36
	30-34	1182	53	461	31
	≥35	345	52	172	23
	Missing	66	N/A	37	N/A

Maternal occupation	Housewife	1764	71	824	33
	Full-time	818	40	385	27
	Part-time	80	93	61	66
	Others	142	105	82	57
	Missing	82	N/A	49	N/A

Family structure	Nuclear	1862	60	857	32
	Extended	869	76	430	40
	Single parent	31	119	28	36
	Missing	124	N/A	86	N/A

Notes: Because of missing data for denominators, ratios may exceed 100%.

### Parity

Parity ≥ 4 was associated with lower response ratios regardless of screening participation status (Table 2).

### Maternal age

Maternal age was inversely associated with response ratios among participants. Maternal age ≥ 35 was a risk factor for low response ratios among nonparticipants (Table 2).

### Maternal occupation

Full-time employment was predictive of lower response ratios, especially for screening participants (Table 2).

### Family structure

Nuclear family status was associated with lower response ratios than extended family status regardless of participation status.

Except for parity, a few percentages of demographic data were missing on the registry, and 60% to 80% of the missing demographic data belong to Kaga City (N= 408). After excluding the Kaga City data, response ratios changed by a few percentage points in most of the strata.

### Self-reported screening participation status compared to the screening registry

Five out of 1,826 screening participants reported not having participated in the screening. Of these five, four mothers were multiparous. In contrast, 21% of nonparticipants replied they had participated in the screening. The incorrect reporting rates varied greatly among the five districts (Table 3). Other factors associated with the proportion of incorrect reporting were lower parity and maternal age (Table 3). Further stratifications were not possible due to the small cell size.

**Table 3.** Percentage of incorrect participation status among nonparticipants by public health district, maternal age and parity, ishikawa prefecture, Japan, 1999.

		N	%
Public Health	Kaga	90	41
Districts	Isikawa-Central	89	26
	Noto-Central	53	28
	Noto-North	25	16
	Kanazawa	201	10
	Total	458	21

Maternal age	<25	70	10
	25-29	189	24
	30-34	144	26
	≥35	39	18

Parity	1	176	26
	2	181	20
	3	81	20
	≥4	20	5

## DISCUSSION

This study demonstrated the usefulness of colour-coded questionnaires to detect response bias in an anonymous survey. This method enabled us to calculate accurate response rates by screening participation status. If the responses on the questionnaire were taken at face value, the response rates would have been 25% for nonparticipants and 66% for participants.

This method also enabled us to calculate the proportion of mothers providing incorrect information regarding screening participation status. It is not clear why some nonparticipants reported to have participated in the screening in the anonymous survey. Since no tracing was used, mothers did not have to respond if they did not wish to participate in the survey. It is unlikely that mothers were confused about screening participation status with their older children’s screening participation because primiparous mothers had the highest percentage of incorrect screening participation status. This problem of incorrect reporting may reflect a strong social desirability bias in a conservative society. Mothers may feel psychological pressure to return the questionnaire in an official survey and may also feel uncomfortable reporting nonparticipation status. In this survey, no questionnaire was returned unanswered, which is a form of refusal reported in the literature^5^^)^.

Response ratios exceeded 100 in some strata, possibly due to missing data for denominators, changes in status, or problems in recalling demographic information. We could not evaluate the accuracy of responses. Nevertheless, patterns of response ratios by demographic characteristics are in the expected direction. Higher parity, older maternal age, and full-time employment status were associated with lower response ratios. These demographic characteristics may reflect the degree of maternal interest in the screening or the priority of the postal survey in their lives.

Previous studies have indicated that educational attainment is associated with response rates^6^^, ^^7^^)^. In Ishikawa Prefecture, over 99% of the population receives a high school education^8^^)^. This survey did not request information regarding maternal education; it was thought that asking about educational status would lower response rates in this population. Regional variations in the percentage of students who receive a college education were not correlated with response rates by region^8^^)^. Kanazawa has the highest percentage of people with a college education, and the response rate for Kanazawa was lower than that for Noto-North, where the percentage of people with advanced degree is much lower than the prefecture average, having the highest response rate^8^^)^. It seems that local culture is a stronger predictor of response rates than educational attainment in Ishikawa Prefecture.

Asch and colleagues conducted a systematic review of postal surveys reported in the medical literature and found that anonymity and financial incentives were not associated with higher response rates^2^^)^. In this study, it was a consensus of the planning committee that maintaining anonymity was important to ensure cooperation in this population. Multivariate analysis of published literature results suggests written reminders with a copy of the questionnaire and telephone reminders were each associated with a response rate about 13% higher than surveys that do not use these tools^2^^)^. This study could not test the effect of reminders to increase response rates because of budget constraint. In any event, a 10% increase in response rate for screening nonparticipants would not have met the goal of the survey, i.e., to explore reasons for nonparticipation.

In summary, colour-coded questionnaires detected severe response bias and social desirability bias in a population-based official survey in Japan. Screening nonparticipants were less likely to respond than screening participants, and 20% of nonparticipants identified themselves as screening participants.

## References

[1] Bessho FÅDIs there a future for neuroblastoma mass screening? Med Pediatr Oncol 1998; 31: 106-110. 9680937 10.1002/(sici)1096-911x(199808)31:2<106::aid-mpo12>3.0.co;2-l

[2] Asch DA, Jedrziewski MK, Christakis NAÅDResponse rates to postal surveys published in medical joumalsÅDJ Clin Epidemiol 1997; 50: 1129-1136. 10.1016/s0895-4356(97)00126-1 9368521

[3] Hoeymans N, Feskens EJ, Van Den Bos GA, Kromhout DÅDNon-response bias in a study of cardiovascular diseases, functional status and self-rated health among elderly menÅDAge Ageing 1998; 27: 35-40. 10.1093/ageing/27.1.35 9504364

[4] Van den Akker M, Buntinx F, Metsemakers JFM, Konottnerus JA. Morbidity in responders and nonresponders in a register-based population survey. Fam Practice 1998; 15: 261-263. 10.1093/fampra/15.3.261 9694185

[5] Alderdice F, Petty T, Johnson A, Macfarlane A. The feasibility of using a postal survey method to assess the health and development of 7 year old children of different birth weight. J Epidemiol Community Health 1998; 52: 439-444. 9799878 10.1136/jech.52.7.439 PMC1756733

[6] Larroque B, Kaminski M, Bouvier Colle MH, Holebcque V. Participation in a mail survey: role of repeated mailings and characteristics of nonrespondents among recent mothers. Paediatr Perinat Epedemiol 1999; 13: 218-233. 10.1046/j.1365-3016.1999.00176.x 10214611

[7] Etter J, Pemeger TV. Analysis of non-response bias in a mailed health survey. J Clin Epdemiol 1997: 50; 1123-1128. 10.1016/s0895-4356(97)00166-2 9368520

[8] Ishikawa Basic School Survey (in Japanese). Ishikawa Kikaku-Kaihatsubu, Ishikawa, Japan, 1999.

